# Life situation and mortality among former forensic psychiatric patients

**DOI:** 10.3389/fpsyt.2026.1868709

**Published:** 2026-07-24

**Authors:** Ebba Noland

**Affiliations:** 1 Department of Social Work, Umeå University, Umeå, Sweden; 2 Region Västernorrland, Sundsvall Forensic Psychiatric Centre, Sundsvall, Sweden

**Keywords:** forensic psychiatry, latent class analysis, mental illness, mortality, post-discharge outcomes, social support

## Abstract

**Introduction:**

Former forensic psychiatric patients experience elevated mortality rates, yet limited research has explored how post-discharge life circumstances influence these outcomes. This study investigates mortality risk across different post-discharge life situation profiles in a national cohort of Swedish forensic psychiatric patients.

**Methods:**

Data were collected from the Swedish National Forensic Psychiatric Register 2009–2018 and linked with multiple national registers, covering a sample of 1, 150 individuals discharged from forensic psychiatric care. Latent class analysis (LCA) identified four distinct subgroups based on post-discharge circumstances: the High support group, the General psychiatric needs group, the Working group, and the Family group. These subgroups varied in psychiatric care use, labor market attachment, social integration, and formal support. Mortality data, including external causes of death, were obtained from the Swedish Cause of Death Register and analyzed in relation to life situation class membership.

**Results:**

A total of 107 individuals (9.3%) died during follow-up, with the highest mortality observed in the General psychiatric needs group. The findings suggest that mortality risk is not evenly distributed across groups, with higher risks observed in subgroups marked by psychiatric and social vulnerability. Substance abuse and further psychiatric inpatient care were prominent characteristics of subgroups with elevated mortality.

**Discussion:**

By highlighting the interplay between clinical, social, and economic factors in the post-discharge life, this study underscores the importance of holistic, context-sensitive approaches to risk assessment and intervention. These insights may inform more targeted and effective strategies to reduce mortality among individuals transitioning out of forensic psychiatric care.

## 1 Introduction

Mortality rates among forensic psychiatric patients in Sweden are high, with 9.4% of all discharged individuals deceased within an average follow-up period of 4.6 years (1). Internationally, mortality rates among discharged forensic psychiatric patients have been found to be high in both absolute and relative terms (2). These findings underscore the need for further investigation, particularly to identify which patients are at the greatest risk of mortality, enabling more targeted and effective preventive interventions.

### 1.1 Forensic psychiatric care in Sweden

To contextualize the current study, it is important to understand how forensic psychiatric care is structured. In Sweden, forensic psychiatric patients constitute a legally rather than purely medically defined group. Individuals convicted of crimes that warrant more than a fine, and who are found to have a severe mental disorder meeting specific medico-legal criteria, are sentenced to compulsory forensic psychiatric care under the Forensic Mental Care Act (SFS 1991:1129).

The most common primary psychiatric diagnosis in the group is schizophrenia and other psychotic disorders (3), although other types of diagnoses such as severe mood disorders, neuropsychiatric disorders and certain personality disorders can also meet these legal requirements. All forensic psychiatric patients initially receive inpatient treatment. In most cases, this care is later transitioned to an outpatient format before definite discharge from all forensic psychiatric services. Discharge decisions are in most cases made by the court. They must take into account the risk of reoffending—specifically, whether the individual is likely to relapse into serious criminal behavior due to their mental disorder. They must also consider whether continued forensic psychiatric care is necessary given the patient’s current mental condition and the patient’s personal circumstances (SFS 1991:1129).

Because discharge is partly based on the individual’s life situation, patients are typically only released to living accommodations that are deemed sustainable. However, as the group is diverse and consists of individuals with different types of mental disorders and needs (4), it follows that there are different types of life situations that typically occur post-discharge. Despite this variation, group-level data suggest that former patients generally receive large amounts of societal support post-discharge (1).

### 1.2 Causes and risk factors for mortality

Previous research on mortality among individuals with mental disorders highlights the significance of factors such as diagnosis type, age, and sex (5). Substance use disorders, in particular, have been consistently linked to increased mortality risk across several large-scale studies (6–8). Thus, a substantial body of research already exists on mortality in psychiatric populations more broadly.

While these general psychiatric findings provide a valuable foundation, the forensic psychiatric population represents a uniquely distinct subset of individuals with mental disorders. For instance, those recently discharged from forensic psychiatric care are demographically specific, with the majority being men in their 40s who have been previously convicted for a crime and spent an extensive period deprived of their liberty within the forensic psychiatric services (1).

In line with this, several studies have directly examined mortality in forensic psychiatric populations. Despite some existing Swedish research on the topic of mortality in forensic psychiatric patients, major knowledge gaps remain. Björk et al. (9) reported that 23% of patients discharged from forensic psychiatric care in Örebro County between 1992 and 2007 died during a mean follow-up period of 9.4 years. However, the small sample size (n = 96) limits the generalizability of these results.

More comprehensive data come from Fazel et al. (7), who used national registry data on 6, 520 individuals discharged from Swedish forensic psychiatric care between 1973 and 2009. They found that 7% of patients died within five years of discharge, and 13% within ten years.

Among male patients in Denmark, 44% of the forensic psychiatric patients and 36% of the matched non-forensic psychiatric patients had died within an average follow-up time of 19 years, compared with 36% of matched non-forensic psychiatric patients (10). In the UK, the risk of death among former forensic psychiatric patients was six times higher than in the general population (11), and the risk of death from both natural and unnatural causes was significantly higher than that of the general population (12).

The reduction in life expectancy among people with mental disorders is well established. A recent meta-analysis demonstrated that mental disorders are associated with significantly reduced life expectancy across diagnostic categories, indicating a transdiagnostic pattern (8). Another meta-analysis on patient outcomes across multiple countries concluded that forensic psychiatric patients also exhibit significantly higher mortality rates than former prisoners (2).

### 1.3 Cause of death

Beyond overall mortality, it is also important to consider the specific causes of death in this population. In the Swedish forensic psychiatric sample examined by Björk et al. (9), the leading causes of death were somatic illnesses (55%) and suicide (30%). Fazel et al. (7) similarly found that 23% of deaths were suicides, with an additional 14% of deaths classified as accidental and 2% as homicides.

In a Nordic context, Ojansuu et al. (13) reported that most deaths among former forensic patients in Finland resulted from somatic illnesses. Additionally, forensic psychiatric patients in Finland have also been found to have a seven-fold increased risk of suicide compared with the general population (14). When considering the Finnish research in a Swedish context, however, caution is needed. The Finnish cohort included only individuals with psychotic disorders (15), while only about half of the Swedish forensic psychiatric population had such diagnoses (1). This limits the direct comparability between the two contexts. Outside the Nordic countries, UK research has shown that 32% of deaths among former forensic psychiatric patients were due to suicide (11). In Scotland, however, no apparent overrepresentation of deaths due to suicide or accidental deaths was found (16).

### 1.4 Risk factors for mortality

In additions to causes of death, a number of risk factors have been identified. A diagnosis of substance use disorder (SUD) has previously been shown to be strongly associated with an elevated risk of mortality in Swedish former patients (7). While the study is robust in scale, its findings may have limited present-day relevance due to significant legislative reforms during the study period. The current framework for Swedish forensic psychiatric care was established in 1991, with substantial reforms to outpatient care introduced in 2008 (26, 27). Nevertheless, SUD has also been linked to increased mortality in both forensic and non-forensic psychiatric populations in Denmark (10). Finnish studies similarly identify substance abuse as a major contributor to elevated mortality, accounting for 16% of all deaths and 64% of accidental deaths.

In the Finnish cohort, shorter durations of forensic psychiatric care were associated with increased mortality (13). More broadly, international research consistently shows that mortality risk among forensic psychiatric patients varies according to demographic and clinical characteristics such as age, diagnosis, gender, and ethnicity (5).

Additional prior studies further suggest that situational factors—rather than diagnoses alone—are closely associated with outcomes such as criminal recidivism and discontinuation of pharmacological treatment (17, 18). Uhrskov Sørensen et al. (10) describes not only medical treatment but also social and psychological support, coordination of private and professional networks and relevant treatment for somatic illness as relevant contributors to the mortality of forensic psychiatric patients. This underscores the need for a more holistic perspective when examining the post-discharge circumstances of forensic psychiatric patients, emphasizing the interplay of life conditions rather than isolated risk factors. This aligns with broader conclusions regarding mortality in individuals with mental disorders, where comprehensive multilevel explanatory models and interventions are deemed necessary (8).

Taken together, this body of research indicates that the lives of former forensic psychiatric patients are shaped by a complex interplay of psychiatric, somatic, social, economic, and criminal factors. Despite this, the specific mechanisms driving the elevated mortality in this population are not yet well understood. Given that forensic and non-forensic psychiatric populations share certain features, most notably the presence of mental disorders, some mortality risks likely overlap. For example, Fazel et al. (7) highlight the prevalence of unhealthy lifestyle behaviors across both groups. However, there are also critical differences. Forensic patients are predominantly male, relatively young at discharge, and often have long histories of compulsory care. Since factors beyond mental disorder have already been shown to influence mortality risk, these distinctive group level characteristics justify focused investigation into the lives and mortality of forensic psychiatric patients specifically.

### 1.5 Life situations after forensic psychiatric care: previous work

In light of the complex and heterogeneous life circumstances of former forensic psychiatric patients, there is a need for a holistic, person-centered approach that captures multiple domains of post-discharge life. Traditional variable-centered methods may obscure meaningful patterns of co-occurring social, clinical, and situational factors, whereas person-centered techniques allow for the identification of more holistic life situation profiles.

Latent class analysis (LCA) is one such person-centered method, based on the assumption that membership in unobserved classes (also referred to as subgroups) can be explained by patterns of indicators (19). In a previous study by the author and colleagues (20), LCA was applied to identify subgroups of post-discharge life situations among former forensic psychiatric patients (n = 1, 150). This approach proved useful for capturing heterogeneity in social integration, psychiatric needs, and structural support, and offers a framework for understanding differential risks across different types of life situations. Four distinct post-discharge life situation profiles among former forensic psychiatric patients were identified:

#### 1.5.1 The high support group (54%)

This largest subgroup was characterized by stable living conditions with supported housing, formalized support structures, and permanent welfare benefits. Members had low levels of psychiatric inpatient care, substance use, and recidivism (<1%), but limited connections to the labor market and rarely lived with a partner or had children.

#### 1.5.2 The general psychiatric needs group (22%)

Individuals in this group experienced significant psychiatric and social challenges, including high rates of inpatient psychiatric care and substance abuse. They frequently received both permanent and temporary welfare benefits and had a high rate of criminal reconviction (43%). Social integration was low, with minimal engagement in the labor market and few living with a partner or having children.

#### 1.5.3 The working group (13%)

This subgroup was distinguished by strong labor market attachment (96%), and most individuals received temporary welfare benefits (99%) rather than permanent benefits. Formalized support and supported housing were rare, as were cohabitation with a partner or having children.

#### 1.5.4 The family group (11%)

Members of this class were primarily defined by family life, most living with a partner (94%) and having children (96%). They commonly had some labor market involvement and received a mix of permanent and temporary welfare benefits. Psychiatric care and formal support were infrequent in this group.

The class labels are descriptive and based on the dominant pattern of indicators within each subgroup. Importantly, “support” in this context refers primarily to formal and structural support (e.g., supported housing and welfare benefits), rather than intensity of psychiatric care. Building on these findings, this paper explores mortality patterns across these life situation subgroups to identify how post-discharge circumstances relate to mortality risk.

Despite these insights, existing research remains insufficient for addressing the specific risks faced by this high-risk population, including elevated mortality from both natural causes and external causes such as suicide and other injuries. More refined ways are needed to distinguish those at the highest risk, allowing for the optimal allocation of resources and preventive strategies. Situational factors have been shown to be associated with other outcomes such as criminal reconviction and discontinuation of pharmacological treatment among former forensic psychiatric patients (17, 18). As such factors have been shown to be relevant to several aspects of the post-discharge lives of this group, they may also be relevant to mortality risk. Investigating potential associations could contribute significantly to more effective risk assessment and intervention planning.

### 1.6 Aim

The aim of this study is to examine mortality among former forensic psychiatric patients in relation to different post-discharge life situations, focusing on both all-cause mortality and mortality from external causes (i.e., deaths resulting from external events such as accidents, violence, or self-harm, rather than internal disease processes).

## 2 Materials and methods

### 2.1 Data

The study utilized retrospective, pre-existing register data. The sample was drawn from the Swedish National Forensic Psychiatric Register (SNFPR) for the period 2009–2018. The SNFPR reported a coverage rate of 86% of all forensic psychiatric patients in Sweden, including data from 24 out of 25 forensic psychiatric units nationwide (21). The register contains information on pre-index crime background variables, variables related to the duration and content of forensic psychiatric care, and variables concerning the situation at the time of discharge from services. A comprehensive database was created by linking SNFPR data with information from the National Patient Register (NPR), the Longitudinal Integrated Database for Health Insurance and Labour Market Studies (LISA), the Register of Interventions According to the Act Concerning Support and Service for Persons with Certain Functional Impairments (the LSS register), and reconviction data from the National Council for Crime Prevention (NCCP).

Data on deaths occurring during the follow-up period were obtained from the Swedish Cause of Death Register. In this register, causes of death are classified according to the International Statistical Classification of Diseases and Related Health Problems, 10th Revision (ICD-10). Within ICD-10, codes V01–Y98 refer to “External causes of morbidity and mortality, “ which include categories such as accidents, intentional self-harm, and events of undetermined intent (e.g., drug overdoses with unclear intent). Previous comparisons with the general population have indicated that external causes of death are more prevalent among forensic psychiatric patients (3). Therefore, mortality due to external causes was analyzed separately.

Inclusion criteria comprised registration in the SNFPR and discharge from forensic psychiatric care between 2009 and 2018. Individuals whose care had ended due to death were excluded. A total of 1, 150 individuals met the inclusion criteria, of whom 938 (82%) were male and 212 (18%) female. The average length of stay, combining both inpatient and outpatient forensic psychiatric care, was 4.9 years (Md = 3.7). Follow-up time started at the time of discharge and all individuals were followed until December 31^st^, 2018, or until death. The duration of follow-up ranged from 4 to 3, 644 days (M = 1, 697; Md = 1, 685). A majority of the individuals (89%) had been sentenced to forensic psychiatric care following a violent offense. Pre-index crime criminal convictions (64%) and substance abuse (56%) were common. The mean age at discharge was 43.6 years (Md = 42).

The study was reviewed and approved by the Regional Ethical Review Board in Umeå (Dnr 2018/222–31) and subsequently by the Swedish National Ethical Review Authority (Dnr 2019–05055), following the reorganization of the Swedish ethical review system from regional boards to a centralized national authority. Access to data from the Swedish National Forensic Psychiatric Register (SNFPR) was granted through the required administrative procedures. The study was conducted in accordance with national legislation and institutional requirements.

### 2.2 Latent class analysis

Subgroup membership was identified through LCA using the software Latent GOLD 6.0, following the approach outlined in a previous paper (20). Briefly, LCA was applied to a set of categorical indicators capturing key domains of post-discharge life, including housing situation, welfare benefit use, psychiatric service utilization, substance use, criminal recidivism, and family and labour market integration. These indicators were selected to reflect multiple dimensions of welfare and social integration relevant to former forensic psychiatric patients. Individuals were assigned to latent classes based on their response patterns across these indicators, resulting in distinct subgroups representing different post-discharge life situations.

In LCA, the values or occurrences of indicator variables are understood as being influenced by individuals’ latent class membership (19). The selected indicators were designed to capture various domains of welfare, drawing on established level-of-living research. These welfare domains (also referred to as welfare areas or welfare dimensions) were adapted to better reflect the specific needs and conditions of particularly vulnerable populations, such as the present sample; for a more detailed account of this adaptation, see Noland et al. (20). The indicators included are shown in **Table 1**.

**Table 1:** Indicators of welfare areas included in the latent class analysis of Noland et al. (2023).

Welfare area	Indicator
Health	Post-discharge inpatient psychiatric care (yes/no)
Housing	Main living accommodation at the time of discharge is supported with staff (yes/no)
Substance abuse	Substance abuse diagnosis post-discharge (yes/no)
Employment	Labor market connection, as defined by being in employment/receiving welfare benefits connected to being close to employment (yes/no)
Economic resources/security of income	Receives any permanent welfare benefit post-discharge (yes/no) Receives any temporary welfare benefit post-discharge (yes/no)
Social relations	Living with partner (yes/no) Having children (yes/no)
Political resources	Formalized support, as defined by having a trustee/limited guardian/facilitator (yes/no)
Criminality	Criminal reconviction (yes/no)

Class formation was guided by the Bayesian Information Criterion (BIC), with consideration also given to the likelihood ratio chi-squared statistic (L^2^), degrees of freedom (df), p-values, and the Entropy R^2^ index. Lower BIC values indicate a better model fit (19). The L^2^ statistic reflects the extent to which associations between variables remain unexplained by the model, while a significant p-value suggests poor absolute model fit (22). Entropy reflects the precision with which the model distinguishes between latent classes (19). Following the criteria outlined by Weller et al. (19), the preferred model was one with a low BIC, an L^2^ value not substantially exceeding the degrees of freedom, a p-value above 0.05, and entropy of at least 0.8. Furthermore, it was considered essential that all indicators demonstrated statistical significance (p <.05), indicating that each contributed meaningfully to the differentiation between clusters (23). Fit statistics are presented in **Table 2**, showing that the chosen model was not the best fitting model. Instead, the chosen model was selected due to its combination of acceptable fit statistics and more distinct clusters, providing a more empirically meaningful explanatory model. The post-discharge factors included in the LCA and their prevalence in the four final classes, or subgroups, of the LCA are presented in **Table 3** along with the prevalence of relevant background factors.

**Table 2:** Indicators of fit for models with one through six latent classes. Reproduced from Noland et al. (2023) under the terms of the CC BY 4.0 license (20).

Classes	LL	BIC(LL)	AIC(LL)	L^2^	df	p-value	Entropy R^2^
1	-6634.1	13345.65	13290.17	2462.05	1135	0.00	1
2	-6221.8	12598.64	12487.67	1637.54	1124	0.00	0.71
3	-6034.0	12300.44	12133.99	1261.86	1113	0.00	0.73
4^b^	-5943.3	12196.60	11974.66	1080.54	1102	0.67	0.77
5^a^	-5851.4	12090.17	11812.75	896.63	1091	1	0.77
6	-5821.9	12108.76	11775.86	837.73	1080	1	0.77

^a^ Best fitting model according to criterion.

^b^ Chosen model.

**Table 3:** Prevalence of background and post-discharge factors in former forensic psychiatric patients in Sweden.

	High support group (*n*=619)	General psychiatric needs group (*n*=254)	The working group (*n*=149)	The family group (*n*=124)	Total (*n*=1146)
Background factors	*n*, %				
Male	500 (80.8)	213 (83.9)	119 (79.9)	104 (83.9)	936 (81.7)
Special court supervision	466 (75.3)	169 (66.8)	111 (74.5)	95 (76.6)	841 (73.4)
Pre-index crime substance abuse	306 (50.7)	202 (80.2)	68 (46.9)	68 (56.7)	644 (57.4)
Pre-index crime criminal conviction	362 (58.5)	210 (82.7)	89 (59.7)	75 (60.5)	736 (64.2)
Presence of psychosis	381 (61.6)	166 (65.4)	78 (52.3)	59 (47.6)	684 (59.7)
Presence of personality disorder	97 (15.7)	53 (20.9)	24 (16.1)	32 (25.8)	206 (18)
Post-discharge factors					
Criminal reconviction	3 (0.5)	108 (42.5)	27 (18.1)	19 (15.3)	157 (13.7)
Inpatient psychiatric care					
No	535 (86.4)	29 (11.4)	81 (54.4)	85 (68.5)	64 (63.7)
Below median	62 (10)	85 (33.5)	34 (22.8)	22 (17.7)	203 (17.7)
Above median	22 (3.6)	140 (55.1)	34 (22.8)	22 (13.7)	203 (18.6)
Post-discharge substance abuse	65 (18.2)	201 (79.1)	49 (32.9)	42 (33.9)	357 (31.2)
Main living accommodation supported by staff	295 (47.7)	82 (94.6)	26 (17.4)	24 (19.4)	427 (37.3)
Labor market connection	13 (2.1)	2 (0.8)	143 (96)	58 (46.8)	217 (18.9)
Living with partner	15 (2.4)	5 (2.0)	9 (6)	116 (93.5)	145 (12.7)
Has children	21 (3.4)	20 (7.9)	21 (14.1)	119 (96)	181 (15.8)
Formalized support	295 (47.7)	82 (32.3)	27 (18.1)	21 (16.9)	425 (37.1)
Permanent welfare benefits	446 (72.1)	243 (95.7)	73 (49)	80 (64.5)	842 (73.5)
Temporary welfare benefits	68 (11)	132 (52)	147 (98.7)	86 (69.4)	433 (37.8)
	*M (Mdn)*	*M (Mdn)*	*M (Mdn)*	*M (Mdn)*	*M (Mdn)*
Age at discharge (years)	47 (46)	42.7 (42.5)	38.1 (37)	35.7 (33)	43.6 (37)
Length of stay (months)	69.6 (53.9)	55.3 (41.6)	39.9 (31.5)	45.5 (37.1)	58.9 (44.6)

As shown in **Table 4**, a total of 107 individuals (9.3%) died during the follow-up period. The highest proportion of deaths was observed in the General psychiatric needs group, where 37 individuals (14.6%) died, followed by the High support group with 57 deaths (9.2%). Mortality was lowest in the Working and Family groups, with 7 (4.7%) and 6 (4.8%) deaths, respectively.

**Table 4:** Prevalence of death within the follow-up period in subgroups of former forensic psychiatric patients.

	High support group (*n*=619) n, % of group	General psychiatric needs group (*n*=254) n, % of group	The working group (*n*=149) n, % of group	The family group (*n*=124) n, % of group	Total (*n*=1146) n, % of group
Deceased within follow-up, all causes	57 (9.2)	37 (14.6)	7 (4.7)	6 (4.8)	107 (9.3)
External causes	6 (1)	15 (5.9)	6 (4)	5 (4)	32 (2.8)

When examining deaths due to external causes (e.g., accidents, self-harm, or events of undetermined intent), 32 individuals (2.8% of the total sample, 30% of those who had died) were classified in this category. The General psychiatric needs group had the highest proportion of such deaths (15 individuals, 5.9%), followed by the Working group (6 individuals, 4.0%) and the Family group (5 individuals, 4.0%). In contrast, only 6 individuals (1.0%) in the High support group died from external causes.

### 2.3 Statistical analysis

All statistical analysis was performed using SPSS version 29. Descriptive statistics, including frequencies and percentages, were used to summarize the data. Chi-square tests were conducted to examine differences in mortality across the various subgroups. An alpha level of 0.05 was applied, and all hypothesis tests were two-tailed. To further explore potential differences between subgroups, pairwise chi-square tests were performed. Given that several pairwise comparisons were conducted, a Bonferroni correction was applied to reduce the risk of Type I error (24). Since six pairwise comparisons were conducted among the four groups, the adjusted alpha level was set to 0.05/6 ≈ 0.008. Following this adjustment, only p-values <.008 were considered statistically significant.

To compare survival and the timing of mortality within the subgroups in the sample, Kaplan-Meier survival analysis was conducted, using the time from discharge to death or the end of follow-up as the survival time. Overall differences between the survival curves of the subgroups were assessed using the log-rank test.

Additionally, Cox proportional hazards regression analyses were performed. The subgroups were included as independent variables, together with demographic and clinical variables that were not included among the indicators used in the classification of subgroups. For the subgroups, the General psychiatric needs group was used as the reference category. The other included variables were sex (male/female), age at discharge (years), length of stay in forensic psychiatric care (months), presence of psychosis (yes/no) and presence of personality disorder (yes/no). The analyses were conducted in three sequential models: Model 1 included subgroups; Model 2 additionally included demographic variables; and Model 3 further included clinical variables related to diagnoses and forensic psychiatric care. Subgroup membership was treated as the primary exposure, and models were specified to first estimate unadjusted differences in mortality between subgroups, followed by adjustment for demographic and clinical covariates.The General psychiatric needs group was used as the reference category, as it represented the subgroup with the highest overall burden of psychiatric and social risk factors and the highest observed mortality, providing a clinically meaningful high-risk comparison.

Identical analyses were conducted separately for all-cause mortality, mortality due to external causes, and mortality due to non-external causes. Time at risk was calculated from discharge from forensic psychiatric care until death or end of follow-up, whichever occurred first. Individuals were censored at the end of the follow-up period. In analyses of cause-specific mortality, deaths from other causes were treated as censored. Hazard ratios (HRs) with 95% confidence intervals (CIs) were calculated. Given the limited number of events for certain outcomes, the number of covariates included in the multivariable models was restricted to reduce the risk of overfitting.

## 3 Results

### 3.1 Mortality rates for individuals in the different latent classes

A chi-square analysis comparing all subgroups revealed significant differences in mortality rates (χ^2^ = 14.97, df = 3, p <.001). To identify which specific subgroup comparisons accounted for these differences, six pairwise chi-square tests were conducted (see **Table 5**). After applying a Bonferroni correction, only the difference between the General psychiatric needs group and the Family group (χ^2^ = 7.82, df = 1, p = .005) and the General psychiatric needs group and the Working group (χ^2^ = 9.40, df = 1, p = .002) remained statistically significant, with the General psychiatric needs group exhibiting a higher mortality rate than both the Working and the Family groups.

**Table 5:** Pairwise chi-square analyses of differences in mortality during the follow-up period in subgroups of former forensic psychiatric patients.

	High support group, n=619, mortality 9.2%	General psychiatric needs group, n=254, mortality 14.6%	The working group, n=149, mortality 4.7%	The family group, n=124, mortality 4.8%
	*p*-value			
High support group, n=619, mortality 9.2%	X	,023	,097	,155
General psychiatric needs group, n=254, mortality 14.6%	X	X	,002*	,005*
The working group, n=149, mortality 4.7%	X	X	X	1,000
The family group, n=124, mortality 4.8%	X	X	X	X

*Significant difference after Bonferroni adjustment, *p*<.008.

### 3.2 Survival

**Figure 1:**
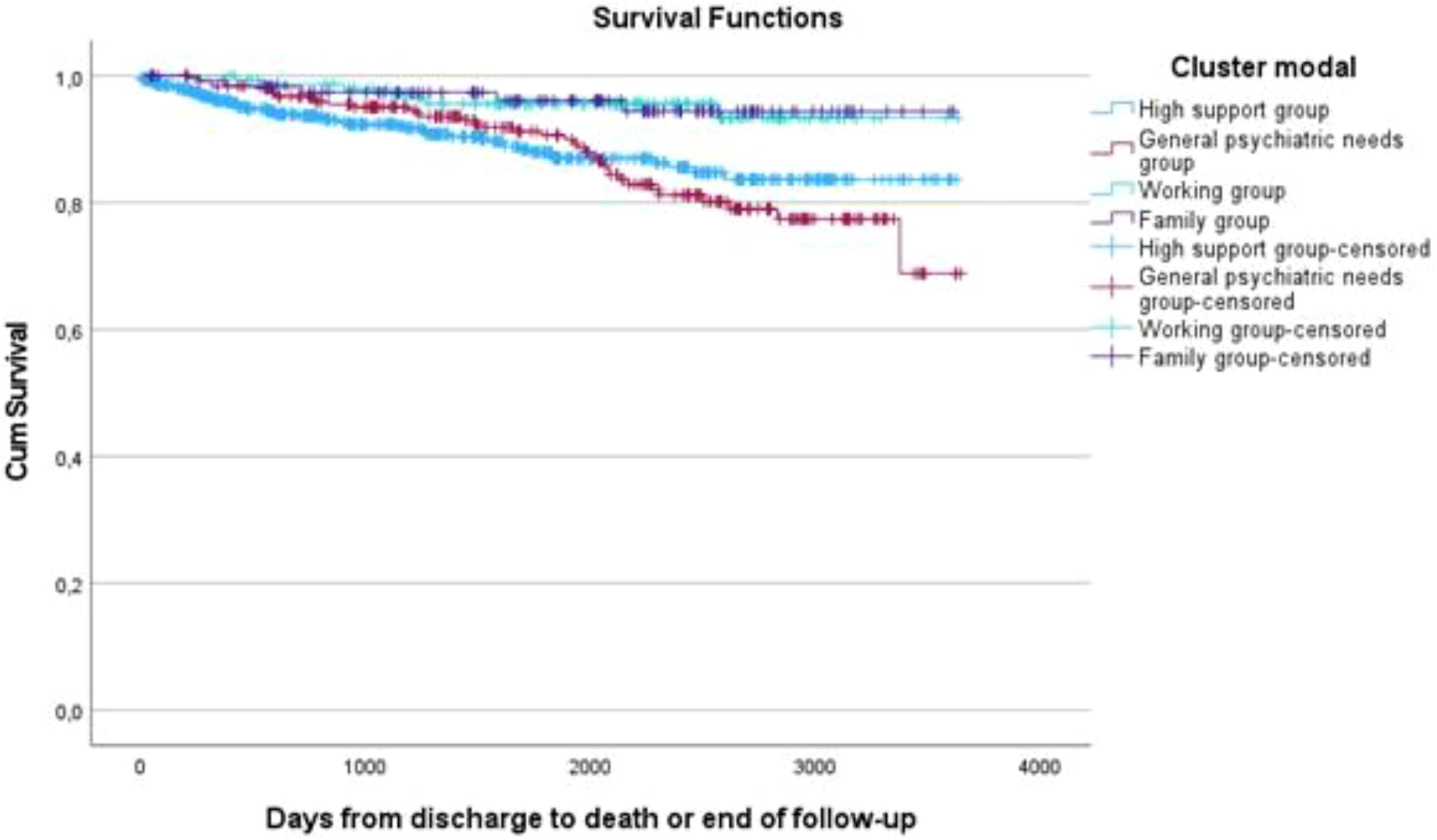
Days from discharge to death or end of follow-up.

In **Figure 1**, the cumulative survival of the different subgroups is shown. Kaplan-Meier analysis was used to estimate the mean survival time in days until death or end of follow-up across the subgroups. The Working group showed the highest mean survival time (M = 3486.08, 95% CI 3382.02, 3590.14), followed closely by the Family group (M = 3475.96, 95% CI 3360.26, 3591.67). The High support group had a mean survival time of 3225.05 days (95% CI [3125.89, 3324.21]), while the General psychiatric needs group had the lowest mean survival time (M = 3187.19, 95% CI 3053.74, 3320.64). The overall mean survival time was estimated at 3286.82 days (95% CI 3222.15, 3351.50), or 9 years and 2 days.

Median survival times could not be estimated for any group due to the high proportion of censored cases.

A log-rank test revealed a statistically significant difference in survival times between the groups, χ^2^ = 16.17, df = 3, p <.001, indicating that time to event differed significantly depending on group membership. Crossing between the survival curves was observed, especially between the High support group and the General psychiatric needs group around the mid-follow-up period. This indicates that differences in survival between some groups varied over time rather than remaining constant.

**Tables 6**-**8** present the results of Cox proportional hazards regression analyses examining all-cause mortality, mortality due to external causes, and mortality due to non-external causes during the follow-up period. Three models were estimated for each outcome, with sequential adjustment for demographic and clinical characteristics.

**Table 6:** Cox regression model: hazard ratio, 95% confidence interval of hazard ratio in square parentheses. Dependent variable: Death (all causes) during the follow-up period (yes/no).

Variable	Model 1	*P*	Model 2	*P*	Model 3	*p*
Post-discharge life situation						
General psychiatric needs(ref.)		.00		.14		.10
High support	.94 [.62-1.42]	.76	.72 [.48-1.1]	.13	.72 [.47-1.11]	.14
Working	.31 [.14-.69]	.00	.48 [.21-1.08]	.08	.45 [.20-1.02]	.06
Family	.29 [.11-.74]	.00	.48 [.19-1.23]	.13	.44 [.17-1.13]	.09
Being male (female ref.)			1.66 [.91-3.03]	.10	1.68 [.92-3.07]	.09
Age at discharge, years			1.06 [1.05-1.08]	.00	1.06 [1.05-1.08]	.00
Length of stay (months)					1.00 [1.00-1.00]	.51
Presence of psychosis					.74 [.50-1.11]	.15
Presence of personality disorder					1.05 [.61-1.83]	.86
-2 Log likelihood	1346.09	.00	1280.18	.00	1277.37	.00
*N*	1142					

**Table 7:** Cox regression model: hazard ratio, 95% confidence interval of hazard ratio in square parentheses. Dependent variable: Death (external causes) during the follow-up period (yes/no).

Variable	Model 1	*p*	Model 2	*p*	Model 3	*p*
Post-discharge life situation						
General psychiatric needs (ref.)		.04		.05		.07
High support	.25 [.10-.65]	.01	.27 [.11-.71]	.01	.30 [.11-.77]	.01
Working	.65 [.25-1.66]	.37	.59 [.23-1.54]	.28	.55 [.21-1.43]	.22
Family	.58 [.19-1.74]	.33	.48 [.16-1.49]	.21	.42 [.14-1.32]	.14
Being male (female ref.)			1.33 [.46-3.80]	.60	1.47 [.51-4.22]	.48
Age at discharge, years			.98 [.95-1.01]	.18	.98 [.95-1.01]	.25
Length of stay (months)					.99 [.98-1.00]	.08
Presence of psychosis					.73 [.34-1.55]	.41
Presence of personality disorder					1.43 [.61-3.38]	.41
-2 Log likelihood	394.59	.03	392.35	.05	387.03	.05
*N*	1142					

**Table 8:** Cox regression model: hazard ratio, 95% confidence interval of hazard ratio in square parentheses. Dependent variable: Death (non-external causes) during the follow-up period (yes/no).

Variable	Model 1	*P*	Model 2	*P*	Model 3	*p*
Post-discharge life situation						
General psychiatric needs (ref.)		.00		.13		.12
High support	1.40 [.84-2.31]	.19	.88 [.52-1.49]	.63	.86 [.51-1.47]	.58
Working	.07 [.01-.55]	.01	.15 [.02-1.15]	.07	.15 [.02-1.12]	.06
Family	.10 [.01-.72]	.02	.19 [.03-1.44]	.11	.19 [.03-1.39]	.10
Being male (female ref.)			1.84 [.88-3.84]	.11	1.80 [.86-3.77]	.12
Age at discharge, years			1.10 [1.08-1.12]	.00	1.10 [1.07-1.12]	.00
Length of stay (months)					1.00 [1.00-1.00]	.74
Presence of psychosis					.81 [.50-1.30]	.38
Presence of personality disorder					.90 [.43-1.89]	.77
-2 Log likelihood	923.78	.00	820.59	.00	819.74	.00
*N*	1142					

#### 3.2.1 All-cause mortality

For all-cause mortality (**Table 6**), post-discharge life situation was associated with mortality in the unadjusted model. Compared with individuals in the General psychiatric needs group, the Working group (HR = 0.31, p <.001) and the Family group (HR = 0.29, p = .01) had lower hazards of death in Model 1. However, these associations were no longer statistically significant after adjustment for sex and age.

In the third and final model, age at discharge was associated with increased mortality risk (Model 3: HR = 1.06, p <.001), while male sex showed a non-significant tendency toward higher mortality. Length of stay and the presence of psychosis or personality disorder were not significantly associated with mortality.

#### 3.2.2 External causes of death

For mortality due to external causes (**Table 7**), the High support group showed a consistently lower hazard of death across all models compared with the General psychiatric needs group (Model 3: HR = 0.30, p = .01). No statistically significant associations were observed for the Working group or Family group.

In contrast to all-cause mortality, age at discharge was not significantly associated with mortality due to external causes. Sex, length of stay, and diagnostic variables were also not significantly associated with this outcome, although length of stay showed a borderline association in the third and final model (HR = 0.99, p = .08).

#### 3.2.3 Non-external causes of death

For mortality due to non-external causes (**Table 8**), membership in the Working group and the Family group was associated with substantially lower hazards of death in Model 1 compared with the General psychiatric needs group. After adjustment for demographic and clinical variables, these associations did not remain statistically significant.

Age at discharge remained a strong predictor of mortality due to non-external causes (Model 3: HR = 1.10, p <.001). Male sex showed a non-significant tendency toward higher mortality, while length of stay and diagnostic variables were not significantly associated with mortality.

In conclusion, age at discharge was strongly associated with mortality from non-external causes but not external causes. Compared with the General psychiatric needs group, the High support group showed a lower risk of mortality from external causes. Post-discharge life situation variables showed associations with mortality in unadjusted analyses that were largely explained by demographic factors after adjustment.

## 4 Discussion

The results show that the subgroups of previous forensic psychiatric patients identified by previous research (20) differ significantly regarding patterns of mortality. In particular, the outcomes are concerning for the General psychiatric needs subgroup. This group displayed the lowest mean survival time, and pairwise comparisons showed that mortality was significantly higher than in both the Working and Family groups. In multivariable analyses, individuals in the High support group showed a lower risk of death due to external causes compared with those in the General psychiatric needs group, even after adjustment for demographic and clinical factors. This subgroup is defined by a life situation including extensive societal interventions, which underscores the need for targeted support beyond what is currently provided.

The contrast is particularly apparent when comparing individuals with general psychiatric needs to those with less extensive support requirements and shorter durations of care. Although individuals in the High support group showed a lower mortality risk than those in the General psychiatric needs group, their outcomes were still less favorable than those observed in the Working and Family groups. This pattern raises questions about both the adequacy and timing of interventions. The crossing of the survival curves between the High support group and the General psychiatric needs group further suggests that mortality risk may not remain constant over time across these subgroups. This pattern may indicate that individuals with different support profiles follow distinct risk trajectories during the follow-up period, with some groups experiencing relatively stable mortality risk while others show an increasing risk of death later in the follow-up period.

Such variation over time also raises questions about whether the timing and continuity of support interventions adequately match changing needs after discharge. The initially observed lower mortality among individuals in the Working and Family groups was weakened after adjustment for age and sex, indicating that demographic differences, and age in particular, may partly explain the survival patterns observed in these groups.

Age at discharge was shown to be a consistent predictor of mortality, particularly for deaths due to non-external causes, while no clear association between age and external causes of death was observed. This suggests that mortality from non-external causes in this population is partly structured by age-related processes. However, this should be interpreted with caution, as previous research indicates that forensic psychiatric populations may experience elevated and premature mortality from natural causes (7). In contrast, mortality from external causes may be more closely related to behavioral or psychosocial risk factors.

While causal conclusions cannot be drawn from this observational data, the results are consistent with previous research linking a history of substance use to increased mortality (13). A pre-index crime history involving substance abuse was common among individuals in the General psychiatric needs group, suggesting that ongoing substance use following discharge may contribute to the mortality differences observed between subgroups. The finding that mortality due to external causes differed between life situation subgroups further supports the possibility that behavioral risk factors, including substance use, may play an important role. Although the sample size limits detailed analysis of the distribution of specific causes of death, external causes accounted for a substantial proportion of deaths within this subgroup.

One notable finding was the relatively low proportion of deaths due to external causes in the High support group, despite this group accounting for a substantial number of deaths overall. This may reflect the combination of extensive structural support, including supported housing and stable welfare provision, together with comparatively low levels of substance use and criminal recidivism observed in this subgroup. It is possible that such support structures contribute to greater stability in daily life, including adherence to treatment, reduced exposure to high-risk environments, and lower engagement in risk-related behaviours (17, 18). In turn, this may reduce the likelihood of external causes of death. Although causal conclusions cannot be drawn, the finding suggests that sustained structural support, particularly among individuals without extensive histories of substance use or criminality, may play a protective role in relation to external mortality risks.

In contrast to findings from a Finnish study indicating that longer length of stay was associated with reduced mortality (15), this association did not reach statistical significance in the present sample, although tendencies in the same direction were observed for deaths due to external causes. This underscores that survival among former patients with extensive psychiatric needs is influenced not only by somatic health, but also significantly by behavioral and social factors. As noted in previous research, comprehensive and multilevel intervention approaches are needed to address lifespan inequalities for people with mental disorders (8).

Interestingly, the excess mortality among individuals in the Working and Family groups was substantially lower in crude analyses, but these differences were not statistically significant after adjustment for demographic variables. This pattern implies that differences in mortality between subgroups may partly reflect underlying differences in age distribution and health status rather than direct protective effects of employment or family living arrangements. It also indicates that mortality in this population may be closely linked to both demographic characteristics and psychosocial functioning. Such factors are likely to be intertwined, for example through the effects of psychotropic medications, unequal healthcare provision, and poorer clinical management of somatic disease among individuals with mental disorders (25).

### 4.1 Strengths and limitations

The present study has several strengths. First, it is based on nationwide register data covering a large cohort of former forensic psychiatric patients, providing high statistical power and minimizing selection bias. The use of linked national registers allowed for comprehensive coverage of multiple domains of post-discharge life. Such register-based designs reduce the risk of recall bias and loss to follow-up. The use of the Swedish Cause of Death Register ensured reliable identification of mortality outcomes.

A further strength is the application of a person-centered analytical approach through LCA. Rather than examining isolated risk factors, this approach enabled the identification of meaningful life situation profiles that reflect the complex interplay between psychiatric, social, and structural conditions. This provides a more holistic understanding of post-discharge circumstances than traditional variable-centered approaches.

However, several limitations should also be acknowledged. First, the observational design does not allow causal interpretation of the findings. While associations between post-discharge life situations and mortality were identified, it cannot be determined whether specific life circumstances directly contributed to mortality outcomes or whether unmeasured underlying factors influenced both subgroup membership and mortality risk. Additionally, subgroup classification was based on post-discharge trajectories and is therefore not a baseline characteristic. This may introduce bias related to differences in follow-up time and potential reverse causality. Accordingly, the subgroup variable should not be interpreted as a baseline exposure in relation to mortality.

Second, the study relied on register-based variables, which, although comprehensive, do not fully capture important aspects of individuals’ lived experiences. For example, the quality of social relationships, the intensity of substance use, and adherence to treatment could not be assessed in detail. Similarly, information on lifestyle-related risk factors was not available, despite their known relevance for mortality among individuals with mental disorders.

Third, the number of deaths due to external causes was relatively small, particularly within some subgroups. This limited the statistical precision of subgroup comparisons and may have reduced the ability to detect additional significant associations. The wide confidence intervals observed in some analyses reflect this limitation. There was also a relatively small number of events for certain outcomes, particularly deaths due to external causes. This limited number of events in relation to the number of covariates included in the multivariable Cox regression models increases the risk of overfitting and may result in reduced precision. The findings from these analyses should therefore be interpreted with caution. Furthermore, visual inspection of the survival curves suggested potential violations of the proportional hazards assumption for some subgroup comparisons, as indicated by crossing curves. This may affect the validity of the Cox regression estimates, and the reported hazard ratios should therefore be interpreted as average effects over the follow-up period.

Finally, the generalizability of the findings is likely to be influenced by the specific characteristics of the Swedish forensic psychiatric system, including its legal framework and extensive welfare support structures. Caution is therefore warranted when generalizing the results to countries with different forensic psychiatric services or social welfare systems.

A key question that remains is how mortality among forensic psychiatric patients compares to that among general psychiatric patients with similar diagnostic profiles. Current evidence does not clarify whether forensic psychiatric care acts as a protective or risk-enhancing factor with regard to mortality in a Swedish context. Structured, matched comparisons with general psychiatric populations are needed to address this issue. Importantly, comparisons between findings from separate studies may be misleading due to demographic differences, particularly in group distributions regarding age and sex (as forensic psychiatric patients are a group consisting largely of relatively young men).

In conclusion, the present study shows that mortality risk among former forensic psychiatric patients is not evenly distributed but varies systematically across distinct post-discharge life situations. Individuals in the General psychiatric needs group appeared particularly vulnerable, with higher mortality and less favorable survival patterns compared with several other subgroups. Differences in mortality due to external causes between subgroups remained after adjustment for demographic and clinical factors. These findings highlight the importance of identifying high-risk subgroups based on broader life circumstances rather than relying solely on clinical characteristics. From a clinical and service perspective, the results suggest that individuals with extensive psychiatric and social needs should be prioritized for sustained follow-up and targeted interventions after discharge. Future research should further examine the mechanisms linking post-discharge life situations to mortality, including the role of substance use and the effectiveness of structured support strategies, to inform the development of more tailored and effective prevention efforts.

## Data Availability

The data analyzed in this study is subject to the following licenses/restrictions: The datasets generated and analyzed during the current study are not publicly available due to Swedish legal and ethical restrictions governing sensitive register data. Access to the data can be granted upon reasonable request and with permission from the relevant Swedish authorities and data custodians. Requests to access these datasets should be directed to rc.datauttag@vgregion.se, https://bestalladata.socialstyrelsen.se/data-for-forskning/, https://www.scb.se/vara-tjanster/bestall-data-och-statistik/.
